# Ethical principles for infodemiology and infoveillance studies concerning infodemic management on social media

**DOI:** 10.3389/fpubh.2023.1130079

**Published:** 2023-03-23

**Authors:** Matheus Lotto, Thokozani Hanjahanja-Phiri, Halyna Padalko, Arlene Oetomo, Zahid Ahmad Butt, Jennifer Boger, Jason Millar, Thiago Cruvinel, Plinio P. Morita

**Affiliations:** ^1^Department of Pediatric Dentistry, Orthodontics, and Public Health, Bauru School of Dentistry, University of São Paulo, Bauru, Brazil; ^2^School of Public Health Sciences, University of Waterloo, Waterloo, ON, Canada; ^3^Department of Systems Design Engineering, University of Waterloo, Waterloo, ON, Canada; ^4^Faculty of Engineering, School of Engineering Design and Teaching Innovation, University of Ottawa, Ottawa, ON, Canada; ^5^Research Institute for Aging, University of Waterloo, Waterloo, ON, Canada; ^6^Centre for Digital Therapeutics, Techna Institute, University Health Network, Toronto, ON, Canada; ^7^Institute of Health Policy, Management, and Evaluation, Dalla Lana School of Public Health, University of Toronto, Toronto, ON, Canada

**Keywords:** health information, infodemic, infoveillance, misinformation, social media

## Abstract

Big data originating from user interactions on social media play an essential role in infodemiology and infoveillance outcomes, supporting the planning and implementation of public health actions. Notably, the extrapolation of these data requires an awareness of different ethical elements. Previous studies have investigated and discussed the adoption of conventional ethical approaches in the contemporary public health digital surveillance space. However, there is a lack of specific ethical guidelines to orient infodemiology and infoveillance studies concerning infodemic on social media, making it challenging to design digital strategies to combat this phenomenon. Hence, it is necessary to explore if traditional ethical pillars can support digital purposes or whether new ones must be proposed since we are confronted with a complex online misinformation scenario. Therefore, this perspective provides an overview of the current scenario of ethics-related issues of infodemiology and infoveillance on social media for infodemic studies.

## Introduction

Social media are web-based interactive communication channels that enable the creation, sharing, and discussion of content by people and online communities (1). There are ~4.59 billion users on these platforms worldwide who interact on everyday topics such as health (2, 3). In this context, social media was a primary source of information on COVID-19 in China at the outset of the pandemic, while four-in-ten Americans considered them essential to follow vaccine-related news (4, 5). Additionally, 81.4% of Saudis users believed that health-related information acquired from social media increased their healthcare awareness, with 73.3% perceiving positive impacts on their health status (6). The literature also shows that many people use these platforms to connect with their peers and exchange their experiences about health conditions (7).

Indeed, the big data originating from these types of user interactions play an essential role in developing infodemiology and infoveillance studies (8, 9). According to Eysenbach (10), “infodemiology is the science of distribution and determinants of information in an electronic medium, specifically the Internet, with the ultimate aim of informing public health and public policy,” while “infoveillance refers to using infodemiology data for digital surveillance purposes.” Although both sciences are essential to support the planning and implementation of public health actions, researchers must make ethical considerations when collecting, analyzing, and presenting digital data derived from people’s activity on social media.

However, the differences in social media data create challenges for experts to adhere to the principles set out by the Declaration of Helsinki (11). For example, acquiring informed consent from each user is unfeasible for large-scale social network datasets, which may contain hundreds of thousands of metadata units (12). As a result, notable aspects concerning informed consent are intangible in social media research, such as the right to withdraw from a study (13). Specifically, it is necessary to propose technics to smooth the discrepancies that emerged from this absence of informed consent since the “participants’ are rarely informed that their data were collected, stored, and analyzed for research purposes. In this sense, researchers can list current studies regarding social network platforms on open data storage to inform communities how their data is being used for public health studies. Additionally, the exponential evolution of social media functionalities exacerbates the difficulties associated with defining ethical research guidelines, which can often be function-specific. Although these concerns motivated several studies to investigate and discuss the adaptation of the conventional ethical approaches to contemporary public health digital surveillance perspectives (14–16), there is a lack of specific ethical guidelines to orient infodemiology and infoveillance studies concerning infodemic on social media, making it challenging to design digital strategies to combat it. Notably, mitigating false or misleading content on social media requires differentiated data treatment since they spread faster than trustworthy ones (17). As a result, it is necessary to clarify if infodemic-related studies’ scope and type of data justify revisions of well-known ethical guidelines regarding digital surveillance, or if their extrapolation is enough to orient investigations in this field.

Infodemic can be defined as an overabundance of information, including false or misleading information, circulating in digital and physical environments during a disease outbreak, such as the COVID-19 pandemic (18). It was the first time that diverse actors employed different communication technologies and social media to inform and connect with people about a common worldwide disease, which generated a massive spreading of content online (19, 20). Although the initial goal of people was to be better informed about COVID-19 toward better health decision-making, the content overload on social media ecosystems hampered users’ selection of trustworthy information (21). Then, misinformation negatively impacted the acceptability of the various COVID-19 vaccines in different countries, contributing to an increased prevalence and severity of cases in some countries (22).

In this context, misinformation is conceptualized as an umbrella term that embraces different types of information disorders, such as misinformation, disinformation, and mal-information (17, 23–27). While misinformation is defined as false informationally-oriented content grounded on truth (25–27), disinformation is intentional false content to purposively harm a person, social group, organization, or country motivated by specific interests, such as social, financial, psychological, and political ones (25–27). Furthermore, mal-information is content based on reality but is used willfully and intentionally to inflict harm on a person, social group, organization, or country. (26). It is noteworthy that despite the divergence in the definitions concerning the author’s intentionality, both can result in adverse consequences for health consumers, e.g., developing and reinforcing damaging beliefs (28).

Therefore, this perspective aimed to provide an overview of the current scenario on ethics-related issues regarding infodemiology and infoveillance, proposing directions for infodemic management studies.

## Ethical aspects concerning digital health studies

The most challenging ethical issue concerning public health is suitably balancing possible risks and harms to people and communities while protecting and promoting their health (29). This challenge also impacts infodemiology and infoveillance social media studies since their ultimate aim is supporting public health outcomes. In fact, principles-based ethics is internationally recognized as a coherent and justified set of moral issues for the field of biomedicine (30, 31). More recently, high-impact systematic reviews used ethical principles to describe the best moral practices involving public health studies on social media, and, thus, supported the present perspective (14, 32). Accordingly, we have presented the five principles of beneficence, nonmaleficence, autonomy, equity, and efficiency, highlighting their respective relevance to infodemic studies below.

### Beneficence

Beneficence is the obligation of health providers to act for the benefit of people based on moral rules, such as charity, mercy, and kindness (33, 34). Therefore, beneficence supports the prevention and control of conditions that cause harm to people (33). Regarding this pillar, infodemiology and infoveillance projects should be designed to promote populational health improvements regarding specific conditions. In this sense, social media interventions must support the healthcare needs of the target population, supporting the improvement of limitations of traditional epidemiological methods, such as extrapolating data generated outside the public health systems, i.e., data that was not originated primarily for epidemiology goals (35). For instance, screening out misinformation promotes beneficence to communities because it facilitates the selection of trustworthy information and, thus, better decision-making concerning current health, social-political, and economic conditions. Furthermore, these strategies may provide advantages to people in different ways, including real-time monitoring of people’s digital activity on specific issues and educational health policies endorsement grounded on users’ behaviors (33, 36–38).

### Nonmaleficence

Nonmaleficence is the responsibility attributed to professionals who do not inflict harm on individuals, resulting in the need to weigh the benefits against the burdens of public health outcomes (33). Indeed, carefully planned and people-centered health outcomes are essential to achieving community trust and developing significant health actions for everyone (39). In this way, the use of non-health data, the stigmatization of risk factors, and the violation of privacy may lead to the mistrust of public health intentions, thus undermining nonmaleficence principles (14). Hence, researchers of misinformation studies should clearly define actions to reduce the potential harms of data collection and analysis, such as adopting a data management plan and restricting their studies to only using publicly available social media data. Notably, previous infodemic-related investigations have presented significant findings using public social networking content (8, 40).

### Autonomy

Autonomy is the state or condition of individuals leading their life according to authentically personal reasons, values, and desires (41). As a result, this principle recognizes people’s right to self-determination and represents the determinants proposed to minimize possible violations (14). Individuals should be allowed to exercise their capacity for self-determination; all people have an intrinsic and unconditional worth that influences their personal and moral choices (42). People often do not expect their data to be employed in public health surveillance since there is no specification for health data reporting in user agreements, even though they cover consent from legal aspects. Nevertheless, some governments are actively implementing initiatives to give users autonomy over their data, such as the European Union General Data Protection Regulation (GDPR), which would allow public health authorities to directly request that people share their social media data when needed (35).

Consequently, anonymizing or aggregating data is fundamental for applying social media data in infodemic-related studies to respect the users’ privacy (14, 43). Although the anonymization process sometimes may not be sufficient to protect the users’ privacy in a social media environment (demonstrating the importance of using public data), it is imperative that researchers remove data and metadata that allow the identification of individuals (16). In parallel, it is possible to aggregate similar social networking publications and present them together, mitigating the identification of the original content. Moreover, users’ authenticity is also essential to ensure a genuine narrative of findings, respecting their autonomy (16). On the other hand, the high prevalence of fake and bots profiles on social network hamper ensuring users’ identities. Specifically, health misinformation is frequently spread by these profiles, denoting the importance of users’ authenticity for infodemic studies. For example, 66% of known bots disclosed COVID-19 information and misinformation on Twitter during the pandemic (44). Interestingly, new authentication user tools emerged as an option to detect automated bots present on social networks (45).

### Equity

Equity is the absence of systematic disparities between groups with different underlying social advantage/disadvantage levels (46). In this regard, most definitions of health equity are based on ethical judgments and commitment to social justice, requiring that a target population be afforded fair, equitable, and appropriate opportunities regarding public health interventions (33, 47). Thus, it is essential to determine whether the short-and long-term benefits and burdens are fairly distributed between different socio-demographic groups (48). Although social media-grounded studies allow researchers and managers to access a huge volume of data and, thus, strategies that involve many users, it is noteworthy that a worldwide population portion still does not have access to home and mobile Internet. In this way, the extrapolation of infodemiology and infoveillance data concerning infodemic for offline measures (e.g., developing health promotion policies and disclosure of educational campaigns) is desirable for covering communities indistinctly (including those without access to the Internet). Further, planning social media studies that tackle health equity involves identifying and acting on the root causes of structural forms of oppression and also investigating health misinformation topics that impact the diverse layers of society differently (48).

### Efficiency

Efficiency is fundamentally based on the ability to measure and assess the improvement of resources, i.e., this pillar is directly related to the cost-effectiveness of digital health systems (14). Certainly, grounding these measures on scientific evidence is necessary since researchers and public health agencies often have limited resources (49). Hence, implementing cost-effective-oriented infodemic control systems requires, (a) applying automated applications to manage and analyze data (focusing on regular maintenance software developer work to prevent the algorithms from becoming obsolete) and (b) designing and implementing misinformation tracking and feasibility studies. Additionally, public health managers should be aware of the continuous updating of these data and propose partnerships with social media companies to avoid the discontinuation of access (50). More importantly, this principle is particularly interesting when extrapolating these digital approaches to developing countries while promoting the democratization of healthcare.

Table 1 summarizes the above-described ethical principles about the infodemic scenario.

**Table 1:** Ethical principles to orient infodemiology and infoveillance studies for infodemic perspectives.

Principles	Short definition	Examples of actions concerning infodemic
*Beneficence*	Obligation of health providers to act for the benefit of people based on moral rules, such as charity, mercy, and kindness. Beneficence supports the prevention and control of conditions that cause harm to people	(1) Real-time monitoring of people’s online activity on specific misinformation issues, endorsing educational health policies based on users’ seeking behavior. (2) Screening out of misinformation on social media, promoting better individual decision-making concerning current health, social-political, and economic conditions.
*Nonmaleficence*	Responsibility attributed to professionals who do not inflict harm on individuals, resulting in the need to weigh the benefits against the burdens of public health outcomes	(1) Collecting only publicly available social media data. (2) Adopting a data management plan to orient the collection and analysis of data.
*Autonomy*	State or condition of individuals leading their life according to authentically personal reasons, values, and desires. Autonomy recognizes people’s right to self-determination and represents the determinants proposed to minimize possible violations	(1) Anonymizing social media data and metadata to preserve users’ privacy. (2) Aggregating similar social media data to avoid the users’ identification.
*Equity*	The absence of systematic health disparities between groups with different underlying social advantage/disadvantage levels. Equity requires that a target population be afforded equal opportunities regarding public health interventions, including fair distribution of the benefits	(1) Extrapolating infodemiology and infoveillance data for offline measures, such as proposing health promotion campaigns and disclosing educational campaigns. (2) Implementing digital health systems accessible for developing and developed countries, propitiating the democratization of misinformation control approaches.
*Efficiency*	It measures and assesses the improvement of resources, relating directly to the cost-effectiveness of digital health systems	(1) Applying automated algorithms to manage and analyze social media data. (2) Developing digital systems and solutions based on misinformation-related characterization, tracking, and feasibility studies.

## Discussion

The five principles of beneficence, nonmaleficence, autonomy, equity, and efficiency can aid the decision-making process of public health authorities and researchers concerning ethics issues on social media in infodemic contexts. Notwithstanding, they should be harmoniously weighted and balanced to achieve effective digital strategies for different communities. Accordingly, the principles must be fulfilled as a *prima facie* obligation unless they conflict with each other in a specific instance (33). Although some of these principles share similar action points (e.g., preserving users’ privacy in the nonmaleficence and autonomy dimensions), the misinformation scenario makes it difficult to employ these ethical points in the same way as for other infodemiology and infoveillance purposes. For instance, the promotion of equity vis-à-vis the viral spreading of misinformation is still a challenge for public health managers, however, their active engagement with the major false or misleading information is necessary to formulate public policies and strategies for disadvantaged communities. To address this dilemma, the World Health Organization recently proposed a deliberation of the issues among a panel of experts, i.e., to discuss the ethical framework and tools for infodemic management (51). Meanwhile, the extrapolation of previously described ethical issues can suffice as a complementary solution to the WHO’s current agenda.

The control of the negative impacts of online misinformation depends on platforms’ cooperative actions with public health authorities, such as screening and removing false or misleading information based on the best scientific evidence (40, 52). Then, companies need to be more transparent about developing their algorithms from users’ activities, concomitantly demonstrating their efforts to prevent the spreading health misinformation. In parallel, health managers and policymakers need to discuss in-depth the ethical and legal implications for potential propagators (users who spread misinformation) and facilitators (social media companies) to formulate regulatory principles that can address this phenomenon more effectively (53).

Simultaneously, it is necessary to clarify the limits of data privacy and freedom of speech in infodemic contexts that have the potential to generate a high humanitarian cost. Personal independence and freedom of speech are highly valued in Western societies and viewed as essential values of free and democratic nations. However, the unlimited perception of the achievement of freedom of speech can cause harm to individuals, communities, and nations, e.g., by promoting drugs or herbals known as ineffective in treating a specific disease only by profit (54, 55). Moreover, users are typically concerned about sharing their private information for digital health purposes due to perceiving implications on insurance coverage, medical care, and data security (56). However, strategies to counter negative infodemic primarily use the information available to the public on social media and only disclose the information anonymously, still safeguarding autonomy.

People must be aware of the importance of sharing their social media information to support the development of strategies to control health misinformation. Thus, data literacy is an essential skill that could be developed during primary and secondary school education in both developing and developed countries. Likewise, other literacies are also necessary to support individuals in consuming trustworthy information on social media and ensure equity between communities through the smoothing out of disparities, such as digital literacy, media literacy, and scientific literacy (57–59). Conversely, low and middle-income countries tend to suffer more prominently from the impacts of online misinformation since the levels of these constructs are usually greater in high-income countries. As a result, the actions involving infodemic management demand more global initiatives. Regrettably, the lack of unified communication about health data between countries and international organizations amplifies the health inequities associated within the infodemic scenario. Notably, a significant role of global health governance (GHG) is to help countries to achieve health equity through managing external threats, stronger international solidarity, and more inclusive guidelines and policies (60). GHG is defined as “the use of formal and informal institutions, rules, and processes by states, intergovernmental organizations, and non-state actors to deal with challenges to health that require cross-border collective action to address effectively” (61). Hence, effective responses to the infodemic require cooperation between states, social media companies, and global health governance to share data and regulate information. Specifically, although many countries have the autonomy and capacity to manage their own health data, ethical guidelines *via* GHG should orient and support the control of misinformation globally.

## Conclusion

Considering the current lack of ethical guidelines for infodemiology and infoveillance research concerning infodemic, the principles presented in this perspective considered the specificities of data acquisition, storage, analysis, and application to contribute to the design and development of health misinformation studies on social media. In light of this perspective, public health authorities, researchers, policymakers, and society should seriously discuss and consider a new ethical framework to cover all details respecting infodemic-related studies.

## Data availability statement

The original contributions presented in the study are included in the article/supplementary material, further inquiries can be directed to the corresponding author.

## Author contributions

ML, TH-P, HP, and AO wrote the first draft of the manuscript. ZB, JB, JM, TC, and PM provided critical review of the manuscript and project conceptualization. All authors contributed to manuscript revision, read, and approved the submitted version.

## Funding

This work was supported by the São Paulo Research Foundation (grant #2021/10732-5).

## Conflict of interest

The authors declare that the research was conducted in the absence of any commercial or financial relationships that could be construed as a potential conflict of interest.

## Publisher’s note

All claims expressed in this article are solely those of the authors and do not necessarily represent those of their affiliated organizations, or those of the publisher, the editors and the reviewers. Any product that may be evaluated in this article, or claim that may be made by its manufacturer, is not guaranteed or endorsed by the publisher.
